# Various Extraction Methods for Obtaining Stilbenes from Grape Cane of *Vitis vinifera* L.

**DOI:** 10.3390/molecules20046093

**Published:** 2015-04-08

**Authors:** Ivo Soural, Naděžda Vrchotová, Jan Tříska, Josef Balík, Štěpán Horník, Petra Cuřínová, Jan Sýkora

**Affiliations:** 1 Department of Post-Harvest Technology of Horticultural Products, Faculty of Horticulture, Mendel University in Brno, Valtická 337, Lednice 69144, Czech Republic; E-Mail: josef.balik@mendelu.cz; 2 Global Change Research Centre, Academy of Sciences of the Czech Republic, v. v. i., Branišovská 31, České Budějovice 37005, Czech Republic; E-Mails: vrchotova.n@czechglobe.cz (N.V.); triska.j@czechglobe.cz (J.T.); 3 Institute of Chemical Process Fundamentals, Academy of Sciences of the Czech Republic, v.v.i., Rozvojová 2/135, 16502 Prague 6, Czech Republic; E-Mails: hornik@icpf.cas.cz (Š.H.); curinova@icpf.cas.cz (P.C.); sykora@icpf.cas.cz (J.S.)

**Keywords:** *Vitis vinifera* L., grape cane, stilbenes, accelerated solvent extraction (ASE), microwave-assisted extraction (MAE), LC-MS

## Abstract

Grape cane, leaves and grape marc are waste products from viticulture, which can be used to obtain secondary stilbene derivatives with high antioxidant value. The presented work compares several extraction methods: maceration at laboratory temperature, extraction at elevated temperature, fluidized-bed extraction, Soxhlet extraction, microwave-assisted extraction, and accelerated solvent extraction. To obtain *trans*-resveratrol, *trans*-ε-viniferin and r2-viniferin from grape cane of the *V. vinifera* variety Cabernet Moravia, various conditions were studied: different solvents, using powdered *versus* cut cane material, different extraction times, and one-step or multiple extractions. The largest concentrations found were 6030 ± 680 µg/g dry weight (d.w.) for *trans*-resveratrol, 2260 ± 90 µg/g d.w. for *trans*-ε-viniferin, and 510 ± 40 µg/g d.w. for r2-viniferin. The highest amounts of stilbenes (8500 ± 1100 µg/g d.w.) were obtained using accelerated solvent extraction in methanol.

## 1. Introduction

Resveratrol has been studied intensively in recent decades as one of the major stilbene derivatives [1,2]. It is believed that this substance bears responsibility for the French paradox connected with wine consumption [3,4]. In addition to resveratrol, the stilbene derivative family encompasses such other biologically active compounds as viniferins [5], piceid [6], pinosylvin [7] and pterostilbene [8], to mention just the most important ones [9]. Wine production and viticulture technology produce four types of waste plant material: grape canes, leaves [10], grape marc and young lateral shoots. According to the literature, stilbenes contained in waste material from wine production are good sources of these substances for the pharmaceutical, cosmetic, and food industries [11]. In order to maximize yields of these compounds from viticulture and wine production byproducts, development of improved methods is required.

It is known from the literature that some of the best solvents for stilbene extraction are alcohols (methanol or ethanol) from the protic group [11]. We studied extraction mainly using methanol as a protic solvent because of its physical properties, and we compared its extraction power with acetone as an aprotic solvent. In some cases we performed additional extraction steps to improve yields, because it has been observed, for example, that from the first extraction of stilbenes from grape cane only approximately 40% of total stilbenes were obtained, while the total yield of stilbenes from the first through fourth extractions was around 95% [12].

Our work focused on studying and comparing various methods of extracting stilbenes from grape canes, including maceration at laboratory temperature, higher temperature extraction, fluidized-bed extraction, Soxhlet extraction, microwave-assisted extraction, and accelerated solvent extraction.

## 2. Results and Discussion

In our experiments, we focused on different extraction methods using various conditions: solvents, sizes of material, temperatures, times, and multiple extractions. In most extractions two solvents were compared—methanol and acetone—for extracting mainly stilbenes: *trans*-resveratrol, *trans*-ε-viniferin (a dimer of *trans-*resveratrol), and r2-viniferin (a tetramer of *trans‑*resveratrol). All three compounds were determined by liquid chromatography using diode array detector/fluorescence detector (DAD/FLD) and low-resolution liquid chromatography–mass spectrometry with ion trap (LCMS-IT) system as described in the Experimental section below. Next to LC-DAD/FLD and low resolution LCMS-IT, both viniferins were identified by high-resolution mass spectrometry (HRMS) and by nuclear magnetic resonance (LC-NMR). The results for all studied parameters are detailed in the following paragraphs.

### 2.1. Absolute Concentration

**Figure 1:**
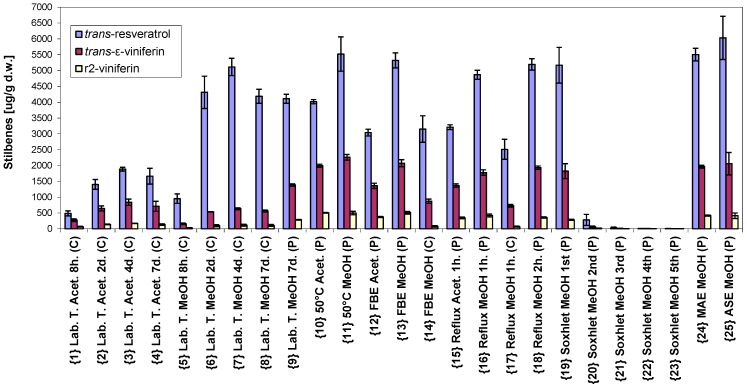
Concentration of *trans*-resveratrol, *trans*-ε-viniferin and r2-viniferin in µg/g dry weight (d.w.) obtained using different extraction procedures (abbreviations are described in Table 1).

The highest values were obtained from grape cane of *Vitis vinifera* cv. Cabernet Moravia were 6030 ± 680 µg/g dry weight (d.w.) of *trans*-resveratol, 2260 ± 90 µg/g d.w. of *trans*-ε-viniferin, and 510 ± 40 µg/g d.w. of r2-viniferin. Overview of the extraction methods is presented in Table 1 and the amount of each above-mentioned stilbene obtained by extraction procedures 1–25 is given in Figure 1. Comparing contents of *trans*-resveratrol and *trans*-ε-viniferin with the literature [11] (3.45 and 1.30 mg/g d.w., cv. Pinot Noir) and [5] (4.7 and 1.7 mg/g dw, cv. Hasaine Sladki), shows a similar ratio of around 2.7 between *trans*-resveratrol and *trans*-ε-viniferin. The highest total concentration of all three stilbenes (8500 ± 1100 µg/g d.w.) was obtained using accelerated solvent extraction in methanol. The higher amount of *trans*-resveratrol in comparison with the two viniferins was from extracting at laboratory temperature from cut grape cane. In that case, the concentration of *trans*-resveratrol was 6.85 times higher than the sum of *trans*-ε-viniferin and r2-viniferin.

**Table 1:** Overview of extraction methods (number, abbreviation and description).

No.	Short Terms (Legends)	Descriptions of Extraction Methods [Type and Temp./Time or Step Extraction/Material/Solvent]
{1}	Lab. T. Acet. 8 h (C)	Laboratory temperature, 8 h, cut, acetone
{2}	Lab. T. Acet. 2 d (C)	Laboratory temperature, 2 days, cut, acetone
{3}	Lab. T. Acet. 4 d (C)	Laboratory temperature, 4 days, cut, acetone
{4}	Lab. T. Acet. 7 d (C)	Laboratory temperature, 7 days, cut, acetone
{5}	Lab. T. MeOH 8 h (C)	Laboratory temperature, 8 h, cut, methanol
{6}	Lab. T. MeOH 2 d (C)	Laboratory temperature, 2 days, cut, methanol
{7}	Lab. T. MeOH 4 d (C)	Laboratory temperature, 4 days, cut, methanol
{8}	Lab. T. MeOH 7 d (C)	Laboratory temperature, 7 days, cut, methanol
{9}	Lab. T. MeOH 7 d (P)	Laboratory temperature, 7 days, powdered, methanol
{10}	50 °C Acet. (P)	Increased temperature 50 °C, 2.75 h, powdered, acetone
{11}	50 °C MeOH (P)	Increased temperature 50 °C, 2.75 h, powdered, methanol
{12}	FBE Acet. (P)	FBE (boiling temperature), 100 min, powdered, acetone
{13}	FBE MeOH (P)	FBE (boiling temperature), 100 min, powdered, methanol
{14}	FBE MeOH (C)	FBE (boiling temperature), 100 min, cut, methanol
{15}	Reflux Acet. 1 h (P)	Reflux (boiling temperature), 1 h, powdered, acetone
{16}	Reflux MeOH 1 h (P)	Reflux (boiling temperature), 1 h, powdered, methanol
{17}	Reflux MeOH 1 h (C)	Reflux (boiling temperature), 1 h, cut, methanol
{18}	Reflux MeOH 2 h (P)	Reflux (boiling temperature), 2 h, powdered, methanol
{19}	Soxhlet MeOH 1st (P)	Soxhlet (boiling temperature), 1st step, powdered, methanol
{20}	Soxhlet MeOH 2nd (P)	Soxhlet (boiling temperature), 2nd step, powdered, methanol
{21}	Soxhlet MeOH 3rd (P)	Soxhlet (boiling temperature), 3rd step, powdered, methanol
{22}	Soxhlet MeOH 4th (P)	Soxhlet (boiling temperature), 4th step, powdered, methanol
{23}	Soxhlet MeOH 5th (P)	Soxhlet (boiling temperature), 5th step, powdered, methanol
{24}	MAE MeOH (P)	MAE (boiling temperature), 30 min, powdered, methanol
{25}	ASE MeOH (P)	ASE (100 °C), 15 min, powdered, methanol

### 2.2. The Highest Yields

**Figure 2:**
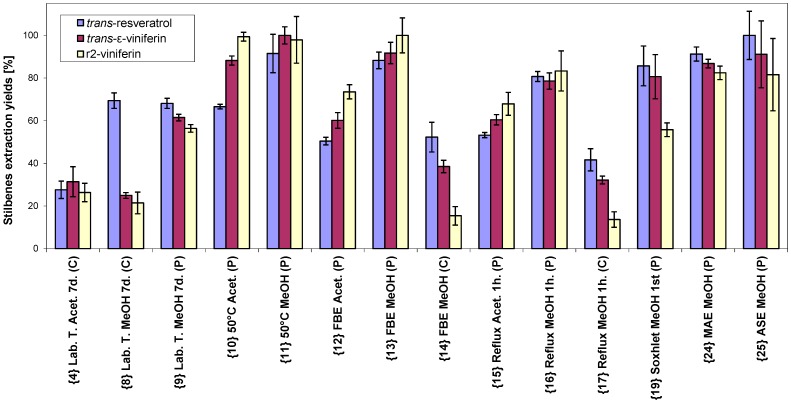
Comparison of extraction yields for stilbenes from different extractions methods (abbreviations are described in Table 1).

The main extraction results are summarized in Figure 2. From the graph, it can be seen that the largest amount of *trans*-resveratrol was obtained by accelerated solvent extraction (ASE) using methanol as solvent and powdered source material, the highest yield for *trans*-ε-viniferin was obtained by extraction at 50 °C, and the highest for r2-viniferin using fluidized-bed extraction with methanol.

If we set 70% as a lowest limit for acceptable extraction yield for all three compounds, then the following extraction methods provide almost the same yield: at 50 °C for methanol and acetone; extraction by ASE; microwave-assisted extraction (MAE); extraction by fluidized-bed extraction with methanol; and extraction with methanol heated to reflux.

### 2.3. The Effect of Conditions on Extraction of Stilbenes

To obtain all three stilbenes from grape cane of the *V. vinifera* variety Cabernet Moravia, various conditions were studied: different solvents, using powdered *versus* cut material, different temperatures, different extraction times, and one-step or multiple extractions.

#### 2.3.1. Solvents

Methanol generally extracted grapevine canes better than acetone in these experiments, and the extraction yield with methanol in percentages of each compound was decreasing in the following order: *trans*-resveratrol, *trans*-ε-viniferin and r2-viniferin. The opposite situation was true for acetone, in which case the percentage values of *trans*-resveratrol were the smallest. The greatest difference between methanol and acetone occurred when extracting of cut grape canes at laboratory temperature; in this case extracts from acetone had very small concentrations of *trans*-resveratrol in comparison with those from methanol, but the contents of *trans*-ε-viniferin and r2-viniferin were a little smaller in methanol than in acetone. A statistical analysis is presented in detail in Table 2.

**Table 2:** Tukey’s honest significance test for stilbenes (*trans*-resveratrol, *trans*-ε-viniferin and r2-viniferin) in µg/g d.w. for different solvents. (Two-way ANOVA: ** *p* < 0.01, * *p* < 0.05, n.s. = not significant. Values are means and standard deviations calculated from three measurements).

Extraction	{1}	{2}	{3}	{4}	{15}	{10}	{12}	{5}	{6}	{7}	{8}	{16}	{11}	{13}
Resveratrol	486.9	1401.9	1884.9	1663.3	3210.8	4016.5	3041.1	951.4	4314.1	5115.4	4189.0	4868.5	5521.6	5324.1
Viniferin	275.2	643.7	838.2	709.5	1366.3	1994.4	1359.1	157.9	536.0	632.1	563.1	1776.7	2260.3	2074.1
r2-Viniferin	67.0	139.8	170.1	133.5	344.7	504.5	373.3	27.2	98.3	115.1	108.7	422.7	496.9	507.5
{1}														
Lab. T. Acet.														
8 h (C)														
{2}	**													
Lab. T. Acet.	**													
2 d (C)	n.s.													
{3}	**	n.s.												
Lab. T. Acet.	**	n.s.												
4 d (C)	*	n.s.												
{4}	**	n.s.	n.s.											
Lab. T. Acet.	**	n.s.	n.s.											
7 d (C)	n.s.	n.s.	n.s.											
{15}	**	**	**	**										
Reflux Acet.	**	**	**	**										
1 h (P)	**	**	**	**										
{10}	**	**	**	**	*									
50 °C Acet.	**	**	**	**	**									
(P)	**	**	**	**	**									
{12}	**	**	**	**	n.s.	**								
FBE Acet.	**	**	**	**	n.s.	**								
(P)	**	**	**	**	n.s.	**								
{5}	n.s.	n.s.	**	n.s.	**	**	**							
Lab. T.	n.s.	**	**	**	**	**	**							
MeOH 8 h (C)	n.s.	**	**	**	**	**	**							
{6}	**	**	**	**	**	n.s.	**	**						
Lab. T.	*	n.s.	**	n.s.	**	**	**	**						
MeOH 2 d (C)	n.s.	n.s.	n.s.	n.s.	**	**	**	n.s.						
{7}	**	**	**	**	**	**	**	**	*					
Lab. T.	**	n.s.	n.s.	n.s.	**	**	**	**	n.s.					
MeOH 4 d (C)	n.s.	n.s.	n.s.	n.s.	**	**	**	*	n.s.					
{8}	**	**	**	**	**	n.s.	**	**	n.s.	**				
Lab. T.	**	n.s.	*	n.s.	**	**	**	**	n.s.	n.s.				
MeOH 7 d (C)	n.s.	n.s.	n.s.	n.s.	**	**	**	n.s.	n.s.	n.s.				
{16}	**	**	**	**	**	*	**	**	n.s.	n.s.	n.s.			
Reflux MeOH	**	**	**	**	**	n.s.	**	**	**	**	**			
1 h (P)	**	**	**	**	n.s.	n.s.	n.s.	**	**	**	**			
{11}	**	**	**	**	**	**	**	**	**	n.s.	**	n.s.		
50 °C MeOH	**	**	**	**	**	*	**	**	**	**	**	**		
(P)	**	**	**	**	**	n.s.	**	**	**	**	**	n.s.		
{13}	**	**	**	**	**	**	**	**	**	n.s.	**	n.s.	n.s.	
FBE MeOH	**	**	**	**	**	n.s.	**	**	**	**	**	**	n.s.	
(P)	**	**	**	**	**	n.s.	**	**	**	**	**	n.s.	n.s.	

#### 2.3.2. Powdered *Versus* Cut Materials

We compared extraction of cut *versus* powdered grape cane at laboratory temperature, by fluidized-bed extraction, and extraction at boiling temperature. Higher yields were always obtained with the extraction of powdered material. In the case of cut cane extracted with methanol, there were large differences in the extraction yields for *trans*-resveratrol, *trans*-ε-viniferin and r2-viniferin.

The amount of *trans*-resveratrol obtained by fluidized-bed extraction of cut material (method 14) was about half of that obtained from powdered material (method 13), but the yield of *trans*-ε-viniferin in method 14 was less than half of that from the powdered material (method 13). The yield of r2-viniferin by fluidized-bed extraction from powdered grape cane (method 13) reached the maximal amount actually contained in the cane (see Figure 2), while the yield of r2-viniferin from cut material (method 14) was only around 15% of that amount. The situations were similar between powdered and cut material when using boiling temperature extraction with methanol (methods 16 and 17, respectively).

More or less the same concentrations of *trans*-resveratrol were obtained by maceration at the laboratory temperature after seven days in powdered (method 9) as in cut material (method 8), but differences between *trans*-ε-viniferin and r2-viniferin yields were more than twofold. A statistical analysis is given in detail in Table 3.

**Table 3:** Tukey’s honest significance test for stilbenes (*trans*-resveratrol, *trans*-ε-viniferin and r2-viniferin) in µg/g d.w. for different particle sizes of grape cane. (Two-way ANOVA: ** *p* < 0.01, * *p* < 0.05, n.s. = not significant. Values are means and standard deviations calculated from three measurements).

Extraction	{14}	{17}	{8}	{13}	{16}	{9}
Resveratrol	3152.3	2511.1	4189.0	5324.1	4868.5	4109.0
Viniferin	869.8	727.3	563.1	2074.1	1776.7	1388.8
r2-viniferin	78.1	69.1	108.7	507.5	422.7	286.2
{14}						
FBE MeOH						
(C)						
{17}	n.s.					
Reflux MeOH	n.s.					
1 h (C)	n.s.					
{8}	**	**				
Lab. T. MeOH	**	n.s.				
7 d (C)	n.s.	n.s.				
{13}	**	**	**			
FBE MeOH	**	**	**			
(P)	**	**	**			
{16}	**	**	n.s.	n.s.		
Reflux MeOH	**	**	**	**		
1 h (P)	**	**	**	*		
{9}	**	**	n.s.	**	*	
Lab. T. MeOH	**	**	**	**	**	
7 d (P)	**	**	**	**	**	

#### 2.3.3. Temperature

Comparison of maceration at laboratory temperature (treatment 9) with extraction at a higher temperature (50 °C, treatment 11) or with other extractions at the boiling point of the solvents (methanol 64.7 °C and acetone 56.5 °C) or with ASE while performing the extraction at 100 °C (treatment 25) shows that *trans*-ε-viniferin and r2-viniferin had smaller yields at laboratory temperature than at higher temperature. Although the yields were similarly lower for the extraction of *trans*-resveratrol at laboratory temperature using acetone (e.g., treatment 4 *versus* treatments 10, 12 and 15), when using methanol, the values for *trans*-resveratrol by maceration at the laboratory temperature (e.g. treatment 8) were nearly the same as at a higher temperature (e.g. treatments 13 and 16). A statistical analysis is given in detail in Table 4.

**Table 4:** Tukey’s honest significance test for stilbenes (*trans*-resveratrol, *trans*-ε-viniferin and r2-viniferin) in µg/g d.w. for different extraction temperatures. (Two-way ANOVA: ** *p* < 0.01, * *p* < 0.05, n.s. = not significant. Values are means and standard deviations calculated from three measurements).

Extraction	{11}	{25}	{9}	{13}	{16}	{19}	{24}
Resveratrol	5521.6	6032.3	4189.0	5324.1	4868.5	5170.5	5505.7
Viniferin	2260.3	2059.8	563.1	2074.1	1776.7	1822.8	1962.5
r2-viniferin	496.9	414.0	108.7	507.5	422.7	282.9	418.4
{11}							
50 °C MeOH							
(P)							
{25}	n.s.						
ASE MeOH	n.s.						
(P)	n.s.						
{9}	*	**					
Lab. T. MeOH	**	**					
7 d (P)	**	**					
{13}	n.s.	n.s.	n.s.				
FBE MeOH	n.s.	n.s.	**				
(P)	n.s.	n.s.	**				
{16}	n.s.	n.s.	n.s.	n.s.			
Reflux MeOH	n.s.	n.s.	**	n.s.			
1 h (P)	n.s.	n.s.	**	n.s.			
{19}	n.s.	n.s.	n.s.	n.s.	n.s.		
Soxhlet MeOH	n.s.	n.s.	**	n.s.	n.s.		
1st (P)	**	n.s.	**	**	*		
{24}	n.s.	n.s.	*	n.s.	n.s.	n.s.	
MAE MeOH	n.s.	n.s.	**	n.s.	n.s.	n.s.	
(P)	n.s.	n.s.	**	n.s.	n.s.	*	

#### 2.3.4. Extraction Time

**Figure 3:**
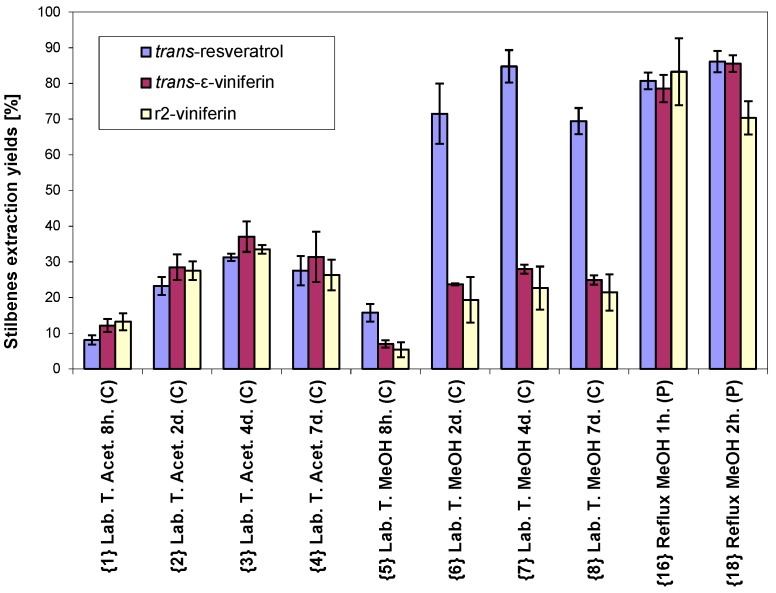
Comparison of stilbenes extraction yields in relation to extraction time (abbreviations are described in Table 1).

Maceration at laboratory temperature and extraction at boiling point temperatures (reflux) were performed for different times. In the case of maceration at laboratory temperature, the time range included 8 h as well as two, four and seven days. The concentrations of all compounds were increasing until day 4, at which the maximum concentration was reached, and the concentrations were slightly decreasing thereafter until day 7. This phenomenon was observed for both solvents, acetone and methanol. In the case of extraction at the boiling point temperature for 1 and 2 h, the contents of *trans*-resveratrol and *trans*-ε-viniferin were slightly increased at the longer boiling time while the concentration of r2-viniferin somewhat decreased. A statistical analysis is given in Table 5 and in detail in Figure 3.

**Table 5:** Tukey’s honest significance test for stilbenes (*trans*-resveratrol, *trans*-ε-viniferin and r2-viniferin) in µg/g d.w. for different extraction times. (Two-way ANOVA: ** *p* < 0.01, * *p* < 0.05, n.s. = not significant. Values are means and standard deviations calculated from three measurements)

Extraction	{5}	{1}	{6}	{2}	{7}	{3}	{8}	{4}
Resveratrol	951.4	486.9	4314.1	1401.9	5115.4	1884.9	4189.0	1663.3
Viniferin	157.9	275.2	536.0	643.7	632.1	838.2	563.1	709.5
r2-viniferin	27.2	67.0	98.3	139.8	115.1	170.1	108.7	133.5
{5}								
Lab. T. MeOH								
8 h (C)								
{1}	n.s.							
Lab. T. Acet.	n.s.							
8 h (C)	n.s.							
{6}	**	**						
Lab. T. MeOH	**	*						
2 d (C)	*	n.s.						
{2}	n.s.	**	**					
Lab. T. Acet.	**	**	n.s.					
2 d (C)	**	*	n.s.					
{7}	**	**	*	**				
Lab. T. MeOH	**	**	n.s.	n.s.				
4 d (C)	**	n.s.	n.s.	n.s.				
{3}	**	**	**	n.s.	**			
Lab. T. Acet.	**	**	**	n.s.	n.s.			
4 d (C)	**	**	*	n.s.	n.s.			
{8}	**	**	n.s.	**	**	**		
Lab. T. MeOH 7	**	**	n.s.	n.s.	n.s.	**		
d (C)	**	n.s.	n.s.	n.s.	n.s.	*		
{4}	*	**	**	n.s.	**	n.s.	**	
Lab. T. Acet.	**	**	n.s.	n.s.	n.s.	n.s.	n.s.	
7 d (C)	**	*	n.s.	n.s.	n.s.	n.s.	n.s.	

#### 2.3.5. Multiple Extractions

**Figure 4:**
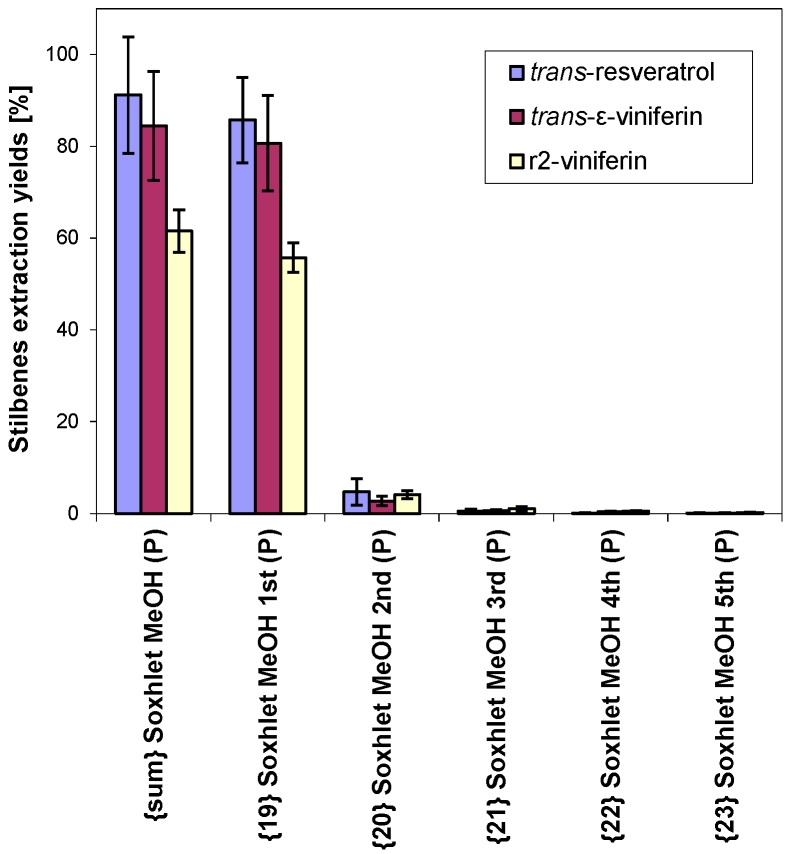
Comparison of stilbenes extraction yields in relation to number of extraction steps (abbreviation {sum} refers to quantity from all five steps together, other abbreviations are described in Table 1).

Multiple extractions were performed by Soxhlet extraction using methanol. The first extraction step (with 10 cycles) had much higher yields compared to the second, third, fourth and fifth steps. The yields of all three compounds by the second step were under 5%, and the yields of the third and further steps were under 1%. A statistical analysis is given in Table 6 and in detail in Figure 4.

**Table 6:** Tukey’s honest significance test for stilbenes (*trans*-resveratrol, *trans*-ε-viniferin and r2-viniferin) in µg/g d.w. for consecutive extraction steps. (Two-way ANOVA: ** *p* < 0.01, * *p* < 0.05, n.s. = not significant. Values are means and standard deviations calculated from three measurements)

Extraction	{19}	{20}	{21}	{22}	{23}
Resveratrol	5170.5	282.2	29.9	8.2	7.7
Viniferin	1822.8	61.5	13.3	8.3	2.8
r2-viniferin	282.9	20.7	5.2	2.5	0.8
{19}					
Soxhlet MeOH					
1st (P)					
{20}	**				
Soxhlet MeOH	**				
2nd (P)	**				
{21}	**	n.s.			
Soxhlet MeOH	**	n.s.			
3rd (P)	**	n.s.			
{22}	**	n.s.	n.s.		
Soxhlet MeOH	**	n.s.	n.s.		
4th (P)	**	n.s.	n.s.		
{23}	**	n.s.	n.s.	n.s.	
Soxhlet MeOH	**	n.s.	n.s.	n.s.	
5th (P)	**	n.s.	n.s.	n.s.	

#### 2.3.6. Extraction Efficiency

Extraction efficiency was influenced by numerous factors—drying, grinding, temperature, extraction time, type of solvent and the extraction procedure (specified in Section 2.3.1, Section 2.3.2, Section 2.3.3 and Section 2.3.4). ASE with methanol and powdered material—extraction method 25 was the most effective treatment (see Table 1).

Extraction method 11 (50 °C, methanol, powdered material), extraction method 13 (FBE, boiling temperature, methanol, powdered material) and also extraction method 24 (MAE, boiling temperature, methanol, powdered material) were comparable with the extraction using ASE.

Extraction of cut material using the same instruments and procedure reached only about 50% as compared with powdered material.

Extraction method 1 (laboratory temperature, acetone, eight hours, cut material) achieved only about 10% efficiency compared to the ASE treatment.

### 2.4. Quantity and Quality of Stilbenes

#### 2.4.1. Determination of Stilbenes

**Figure 5:**
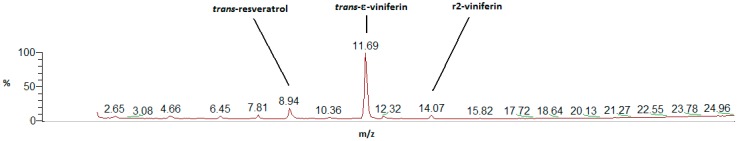
Chromatogram of all three stilbenes (extraction No. 11) in full-scan (positive mode at atmospheric pressure chemical ionization—APCI).

Only three main peaks of resveratrol and its derivatives having fluorescence were analyzed by HPLC-DAD/FLD at 220 nm and low resolution LC-DAD/MS (see Figure 5). Next to these method HRMS (High Resolution Mass Spectrometry) and LC-NMR for identification of both viniferins were used.

In the samples measured by LC-MS, there is a minor peak at the retention time of 12.32 min with *m/z* = 681 and with small fluorescence in the HPLC-DAD/FLD system. This compound could be one of the resveratrol trimers (e.g. amurensin B, amurensin G or gnetin H).

#### 2.4.2. Identification of Viniferins

In contrast to *trans-*resveratrol whose standard was available, *trans-*ε-viniferin (MW = 454, t_R_ = 11.69 min) and r2-viniferin (MW = 906, t_R_ = 14.07 min) were identified by HRMS and LC-NMR. Monoisotopic masses of molecular ions and fragment ions for both viniferins from high-resolution mass spectrometry measurements are given in the Table 7.

**Table 7:** Monoisotopic masses for viniferins in Daltons (full-scan and MS/MS mode)

*trans*-ε-Viniferin C_28_H_22_O_6_			r2-Viniferin C_56_H_42_O_12_		
Formula	Found [*m/z*]	Calculated [*m/z*]	Formula	Found [*m/z*]	Calculated [*m/z*]
C_28_H_23_O_6_^+^ [M+H]^+^	455.1482	455.1489	C_56_H_43_O_12_^+^ [M+H]^+^	907.2745	907.2749
C_28_H_21_O_5_^+^ [M−H_2_O)^+^	437.1373	437.1383	[M+Na]^+^	929.2960	929.2968
C_22_H_17_O_5_^+^ [M−C_6_H_5_O]^+^	361.0740	361.070	[M+K]^+^	945.2305	945.2307
C_13_H_11_O_3_^+^	215.0709	215.0702	C_35_H_27_O_7_^+^	559.1709	559.1751
			C_28_H_21_O_6_^+^	453.1339	453.1332
			C_22_H_17_O_5_^+^	361.1038	361.1070
			C_13_H_11_O_3_^+^	215.0690	215.0702

The obtained UV spectrum of *trans*-ε-viniferin and r2-viniferin in the water-acetonitrile mixture acidified with 0.1% of *o*-phosphoric acid revealed the following maxima: *trans*-ε-viniferin—λ_max_: 226 and 324 nm; r2-viniferin—λ_max_: 226 and 326 nm. Ha *et al.* [13] have reported UV spectrum of *trans*-ε-viniferin in methanol (λ_max_: 226, 285 and 322 nm) and for r2-viniferin in methanol (λ_max_: 224, 285 and 327 nm).

*NMR identification*: The structure elucidation of *trans-*ε-viniferin and r2-viniferin was based on ^1^H and COSY 2D experiment and comparison of literature data [14,15]. The ^13^C-NMR experiment could not be performed (neither direct nor indirect) to further confirm compound structure due to low concentration. Therefore, the assignment of NMR signals was confronted with the predicted ^1^H-NMR spectrum using current version of NMR predictor software [16] and showed good agreement. Some signals were hidden under signal of water; these signals were located from COSY spectrum. The ultimate confirmation of the structure was provided by off-line HRMS performed on trapped chromatographic peaks.

*trans*-ε-Viniferin: ^1^H-NMR: δ (ppm) 7.18 (d, 2H, H-2, H-6, *J*_2,3_ = *J*_5,6_ = 8.5 Hz) 7.15 (d, 2H, H-2', H-6', *J*_2',3'_ = *J*_5,6'_ = 8.6 Hz), 6.92 (d, 1H, H-8', *J*_7',8'_ = 16.4 Hz), 6.82 (d, 2H, H-3, H-5, *J*_2,3_ = *J*_5,6_ = 8.5 Hz), 6.74 (d, 2H, H-3', H-5', *J*_2',3'_ = *J*_6',5'_ = 8.6 Hz), 6.68 (d, 1H, H-14', *J*_12',14'_ = 1.8 Hz), 6.62 (d, 1H, H-7', *J*_7',8'_ = 16.4 Hz), 6.33 (d, 1H, H-12', *J*_12',14'_ = 1.8 Hz), 6.18 (d, 2H, H-10, H-14, *J*_10,12_ = *J*_12,14_ = 1.9 Hz), 6.16 (t, 1H, H-12, *J*_10,12_ = *J*_12,14_ = 1.9 Hz), 5.47 (d, 1H, H-7, *J*_7,8_ = 6.0 Hz), 4.47 (d, 1H, H-8, *J*_7,8_ = 6.0 Hz).

r2-Viniferin: ^1^H-NMR: δ (ppm) 7.20 (d, 2H, H-2, H-6, *J*_2,3_ = *J*_5,6_ = 8.6 Hz) 7.18 (d, 2H, H-2''', H-6''', *J*_2''',3'''_ = *J*_5''',6'''_ = 8.6 Hz), 7.10 (d, 1H, H-6', *J*_5',6'_ = 8.5 Hz), 6.83 (d, 2H, H-3, H-5, *J*_2,3_ = *J*_5,6_ = 8.6 Hz), 6.81 (d, 2H, H-3''', H-5''', *J*_2''',3'''_ = *J*_6''',5'''_ = 8.6 Hz), 6.79 (d, 1H, H-8', *J*_7',8'_ = 16.2 Hz), 6.78 (s, 1H, H-2'), 6.77 (d, 1H, H-5', *J*_5',6'_ = 8.5 Hz), 6.64 (d, 2H, H-2'', H-6'', *J*_2'',3''_ = *J*_5'',6''_ = 8.6 Hz), 6.61 (d, 1H, H-14', *J*_12',14'_ = 1.9 Hz), 6.57 (d, 2H, H-3'', H-5'', *J*_2'',3''_ = *J*_5'',6''_ = 8.6 Hz), 6.55 (d, 1H, H-7', *J*_7',8'_ = 16.2 Hz), 6.34 (d, 1H, H-12', *J*_12',14'_ = 1.9 Hz), 6.31 (d, 1H, H-14''', *J*_12''',14'''_ = 2.0 Hz), 6.12 (d, 1H, H-14'', *J*_12'',14''_ = 2.1 Hz), 6.11 (d, 1H, H-12''', *J*_12''',14'''_ = 2.0 Hz), 6.10 (d, 1H, H-12'', *J*_12'',14''_ = 2.1 Hz), 6.01 (t, 1H, H-12, *J*_10,12_ = *J*_12,14_ = 1.9 Hz), 5.95 (d, 2H, H-10, H-14, *J*_10,12_ = *J*_12,14_ = 1.9 Hz), 5.50 (d, 1H, H-7''', *J*_7''',8'''_ = 5.5 Hz), 5.45 (d, 1H, H-7'', *J*_7'',8''_ = 5.5 Hz), 5.38 (d, 1H, H-7, *J*_7,8_ = 5.5 Hz), 4.46 (d, 1H, H-8'', *J*_7'',8''_ = 5.5 Hz), 4.46 (d, 1H, H-8, *J*_7,8_ = 5.5 Hz), 4.25 (d, 1H, H-8''', *J*_7''',8'''_ = 5.5 Hz).

## 3. Experimental Section

### 3.1. Standards and Solvents

Standard of trans-resveratrol was purchased from (Sigma-Aldrich, Prague, Czech Republic) and measured as solution in pure methanol. Solvents were obtained as follows: methanol, acetone and acetonitrile (Merck, Prague, Czech Republic, LiChrosolv, gradient grade for LC), acetonitrile (Fisher Scientific, Pardubice, Czech Republic, Optima LC/MS), *ortho*-phosporic acid (Fluka, Prague, Czech Republic, p.a.) and formic acid (Sigma-Aldrich, Prague, Czech Republic, gradient grade).

### 3.2. Samples Analysis

#### 3.2.1. High-Performance Liquid Chromatography 

The samples were analyzed using an HP 1050 (Ti-series) HPLC instrument (Hewlett Packard, Palo Alto, CA, USA) on a 3 µm, 150 mm × 2 mm, Luna C18(2) column (Phenomenex, Torrance, CA, USA) with water-acetonitrile-*o-*phosphoric acid mobile phase. Mobile phase A used 5% of acetonitrile +0.1% of *o*-phosphoric acid; mobile phase B used 80% of acetonitrile +0.1% of *o*-phosphoric acid (in vol.%). The gradient was increased from 20% of B to 80% of B during 20 min and from 80% of B to 100% of B during 5 min. Flow rate was 0.250 mL/min and column temperature 25 °C. Injection volume was 5 µL. Also used were an G1315B diode array detector (DAD, Agilent, Prague, Czech Republic) with detection wavelengths at 220 and 315 nm and scanning range 190–600 nm, as well as an G1321A fluorescence detector (FLD, Agilent, Prague, Czech Republic) with excitation wavelength 315 nm, emission wavelength 395 nm, and scanning of emission in the range of 300–600 nm. Finally, the method was validated in terms of linearity, limits of detection, and repeatability [17,18].

#### 3.2.2. Liquid Chromatography–Mass Spectrometry (LC-MS)

Low-resolution LC-MS measurement was performed using an LCQ Accela Fleet (Thermo Fisher Scientific, San Jose, CA, USA) equipped with electro-spray (ESI), atmospheric pressure chemical (APCI), and atmospheric pressure photo (APPI) ionization sources and a photodiode array detector. A 3 µm, 150 mm × 2 mm, Luna C18(2) column (Phenomenex, Torrance, CA, USA) was used with water-acetonitrile-formic acid mobile phase. Mobile phase A used 5% of acetonitrile +0.1% of formic acid; mobile phase B used 80% of acetonitrile +0.1% of formic acid (in vol.%). The gradient was increased from 20% of B to 80% of B during 20 min and from 80% of B to 100% of B during 5 min. Injection volume was 10 µL and flow rate 0.400 mL/min. APCI capillary temperature was 275 °C, APCI vaporizer temperature 400 °C, sheath gas flow 58 L/min, auxiliary gas flow 10 L/min, source voltage 6 kV, source current 5 µA, and capillary voltage 10 V [17,18].

#### 3.2.3. Liquid Chromatography– Nuclear Magnetic Resonance (LC-NMR)

A commercial HPLC system (Dionex UltiMate 3000, Thermo Fisher Scientific, San Jose, CA, USA) with 250 mm × 4.6 mm HPLC column (Luna C18(2), Phenomenex, 5 µm particles, 100 Å pore size) was employed. Fifty microliters of the concentrated methanol solution was injected into HPLC. The separation was done by gradient method described in the HPLC section (see Section 3.2.2.; system acetonitrile–D_2_O) and was monitored at 220 nm and by on-flow ^1^H-NMR detection. ^1^H-NMR observations were conducted on Varian INOVA 500 MHz spectrometer (Varian Inc., Palo Alto, CA, USA) equipped with HCN triple resonance (60 µL active volume) microflow probe. Standard NMR software VnmrJ 4.2 was used. All the separations and NMR detection were conducted at ambient temperature (22 °C). The ^1^H-NMR data were collected in on-flow mode employing WET multiple frequency solvent suppression [19]. The signal of acetonitrile solvent (δ = 2.00 ppm) was suppressed using one scout scan prior to whole data collection. The data acquisition during 1 s acquisition time covering the spectral width of 6 kHz followed after 90° RF pulse (3.4 µs), four transients were accumulated in each spectrum. No relaxation delay was employed.

**Figure 6:**
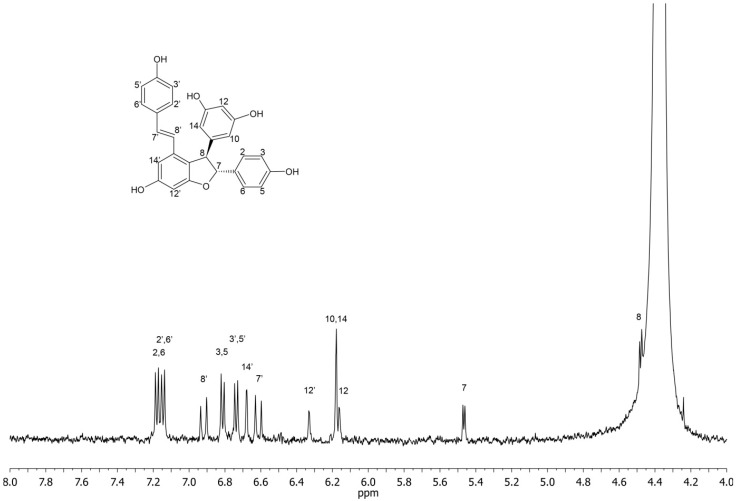
^1^H-NMR spectrum and signal assignment of *trans*-ε-viniferin.

**Figure 7:**
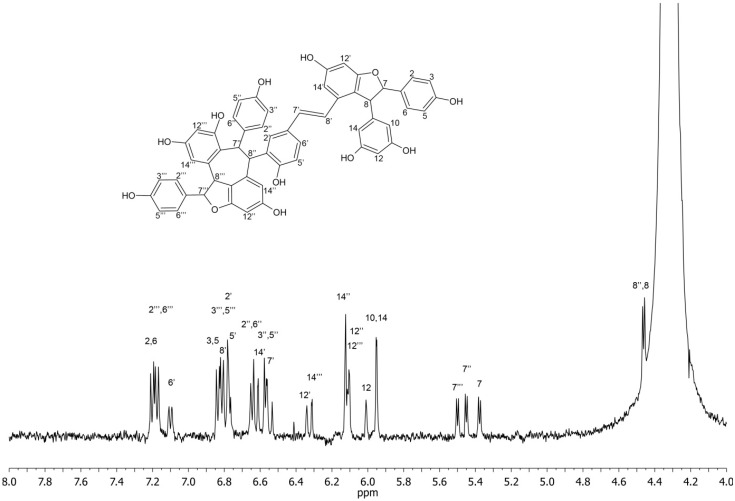
^1^H-NMR spectrum and signal assignment of r2-viniferin. Signal of H-8''' is hidden under water signal at 4.25 ppm.

The detailed analysis of chromatographic peaks was performed in the stop-flow mode. ^1^H-NMR spectra were accumulated over 128 scans (acquisition time 2 s, relaxation delay 1s). COSY experiment took 2 h for *trans*-ε-viniferin and 12 hours for r2-viniferin. The ^1^H-NMR spectra with assigned structures of *trans*-ε-viniferin and r2-viniferin are depicted in the Figure 6 and Figure 7.

#### 3.2.4. High Resolution Mass Spectrometry (HRMS)

The HRMS spectra of given samples were measured at Bruker MicrOTOF-QIII apparatus. Due to the structure of targeted compounds, the ESI source in the positive mode was used and the parameters of the measurement were adjusted as follows: the capillary voltage was 4200 V, the end plate offset was −500 V. The collision cell RF was 350 Vpp. Nitrogen was used as the nebulizer gas (at the pressure of 1.6 bar), just as the drying gas (heated to 180 °C, with the flow of 8 L/min). The scans of MS spectra were carried out in the mass range of *m/z* 80–1550. For the HRMS measurement, the calibration on Na-formate clusters was used.

The samples were delivered by the direct infusion using the syringe pump (Kd-Scientific, KDS-100-CE, 0.5 mL Hamilton syringe, flow 180 mL/min) coupled to the MicrOTOF-QIII mass spectrometer.

For the MS/MS measurements, the precursor ions were isolated by the collision cell energy 2 eV in the window of 2 units (*m/z*) and the product ions were obtained at the collision cell energy rising from 25 to 35 eV, depending on the compound examined.

### 3.3. Samples

Samples were from *Vitis vinifera* L. cv. Cabernet Moravia (crossing: Cabernet Franc x Zweigeltrebe) grown in wine region Moravia, sub-region Slovacko, village Kostice (GPS: 48°44′50″N 16°58′26″E). Grape canes were dried at room temperature for 2.5 months after pruning (March 2013) in darkness and lyophilization was used for final drying. Dried grape canes were then cut to length around 1.0 cm and one part of the canes was powdered in a knife grinder to an approximate size of 1 mm. In this way, we obtained two types of plant samples with different particle sizes.

### 3.4. Extraction Method

We compared the following seven types of extraction methods in our experiments, for both powdered (>1 mm) and cut (~1 cm) material: (1) at laboratory temperature, (2) at 50 °C, (3) at boiling point with a reflux condenser, (4) by fluidized-bed extraction, (5) by Soxhlet extraction, (6) by microwave-assisted extraction (MAE), and (7) by accelerated solvent extraction (ASE).

#### 3.4.1. Extraction at Laboratory Temperature

We added 20 mL of solvent (acetone or methanol) to 2 g of dry grape canes in a flask for use in measuring stilbenes content after four consecutive extraction times. We later took four times 200 µL samples of the material prepared using cut dry canes (not that from using powered canes) after each of 8 h, 2 days and 4 days of extraction. The samples were immediately stored in a refrigerator until analysis. Extractions were stopped on day 7 for both solvents for cut material. Powdered material was done similarly, but measured only one time on day 7 (without precious times: 8 h, 2 and 4 days). Solid portions were then separated by filtration, washed two times with 1 mL of solvent and then solvents were added to bring the final volume back to 20 mL. Extractions were done at temperature was 22 ± 1°C.

#### 3.4.2. Extraction at 50 °C

We used 0.25 g of powdered grape canes and 3 mL of solvent for extraction at 50 °C during 165 min. Smaller amounts of sample and solvent were used than in the laboratory temperature extraction but with roughly the same extraction ratio. After extraction, the mixture was centrifuged at 3500 rpm for 10 min at 20 °C. The supernatant was transferred into a calibrated tube, 1 mL of fresh solvent was added to the solid residue, mixed and this new mixture was centrifuged again. This rinse procedure was then repeated. As a result, the sample was extracted only once and the residue was washed two times with solvent. All supernatants were combined and the final volume of supernatant mixture was adjusted to 5 mL.

#### 3.4.3. Extraction with Solvent Heated to Reflux

The quantity, 1.50 g of grape canes, was transferred into a flask equipped with a reflux condenser and then 50 mL of methanol or acetone was added. The mixture was then heated to reflux at 70 °C in the water bath for 1 or 2 h. The solution was filtered and then 1 mL of solvent was added into the flask and filtration was performed again through the same filter. One milliliter of solvent was used for washing (and then 2 mL again). Final volumes were adjusted to 50 mL. In this way, we obtained solutions in acetone and methanol from powdered source material and a solution only in methanol from the cut source material.

One milliliter from each of the acetone samples (from powdered material) was transferred into a calibrated tube. The acetone solution was dried by a flow of nitrogen gas at 45 °C, after which 1 mL of methanol was added to the dry residue for the analysis.

#### 3.4.4. Fluidized-Bed Extraction

On each frit of an IKA fluidized-bed extractor’s upper part (four vessels in total) we placed 1.5 g of grape cane material and 100 mL of solvent was placed into the bottom flask. The apparatus was heated to 140 °C for 20 min, during which solvent vapors passed through the frit and became liquid on the condenser. Solvent from the condenser dripped onto the sample of grape canes. During extraction, new solvent vapor passed through the sample and in this manner increased its agitation. The cooling process was started after 20 min until the temperature had been reduced to 30 °C. During this period, only the supernatant from the dispersed sample returned to the flask due to the lower pressure resulting from cooling of the solvent. Extraction in the fluidized-bed went through 5 cycles and final volumes were adjusted to 100 mL. In this manner, we obtained methanol samples from powdered and from cut source material and acetone samples only from powdered source material.

#### 3.4.5. Soxhlet Extraction

We placed 1.5 g of grape canes into the thimble in the extractor and 100 mL of methanol in the flask (acetone was not used), volume in Soxhlet apparatus was 50 mL. We performed 10 cycles for each extraction step. One cycle means the time when the volume of supernatant in the thimble goes back into the flask. Completing these 10 cycles took a total time of around 2–3 h. We repeated this step with 10 cycles five times, then removed the supernatant for analysis and added 100 mL of new methanol for the new step. We performed five extraction steps for estimation of the total yield of selected stilbenes. For the sake of good detection and quantification, final volumes from this 5-step extraction were adjusted to 100 mL for the first two steps and to 3 mL for the last three steps.

#### 3.4.6. Microwave-Assisted Extraction (MAE)

Using a 150 W MARS 6 Microwave Reaction System (CEM, Matthews, NC, USA), 1.5 g of powdered grape canes and 50 mL of methanol were placed into the cartridges and extracted. Ramp time was 20 min and hold time was 10 min. After extraction, final volumes were adjusted back to 50 mL.

#### 3.4.7. Accelerated Solvent Extraction (ASE)

Extractions at high pressure 10–10.5 MPa (1450–1520 psi, respectively) and temperature of 100 °C were performed with 0.316 g, 0.386 g and 0.383 g of grape cane (powdered source material) in the ASE 350 Accelerated Solvent Extractor (Thermo Scientific, Dionex). Three 5 min cycles were used for methanol and the final volumes were 6.7 mL, 6.8 mL and 6.6 mL for analyses.

All types of measurements were performed with three parallel samples for statistical evaluation. All samples after extraction were immediately placed into a refrigerator.

### 3.5. Statistical Analysis

All extracts of stilbenes were measured three times. Averages, standard deviations and *p* < 0.01 or *p* < 0.05 (significant *vs.* non-significant differences) were calculated and detailed in Table 1, Table 2, Table 3, Table 4, Table 5 and Table 6 and Figure 1, Figure 2, Figure 3 and Figure 4. The data given in these tables were analyzed by ANOVA, applying the Tukey multiple range test for making comparisons with Statistica Cz 12 and MS Excel 2010 software.

## 4. Conclusions

This work compared various extraction methods for obtaining viniferins and *trans*-resveratrol from grape cane of *Vitis vinifera* L. cv. Cabernet Moravia. The results show that the best extraction method for *trans*-resveratrol is accelerated solvent extraction using methanol and providing 6030 ± 680 µg/g d.w. of *trans*-resveratrol it could be caused not only by temperature effect but also by pressure effect. Higher temperature extraction is better for *trans*-ε-viniferin with the yield of 2260 ± 90 µg/g d.w. of that compound. For the last eluting stilbene, r2-viniferin, the maximum amount of 510 ± 40 µg/g d.w. was obtained by fluidized-bed extraction. As expected, higher yields were obtained from the extractions with powdered source material than with cut material. There was a large difference in yields between *trans*-resveratrol (>80%) and *trans*-ε-viniferin (<30%) at laboratory temperature in the case of cut grape cane. This difference could be used for the separation and concentration of resveratrol. Next to the size of material, regarding extraction yield, multiple extraction has also afforded higher yield compared with single step extraction; similarly extraction at higher temperature is better than at laboratory temperature; in case of time, some maxima of yields were observed (four days at laboratory temperature for both solvents), but generally methanol was the better extraction solvent than acetone. The structure of obtained *trans-*ε-viniferin and r2-viniferin was confirmed by NMR and high resolution mass spectrometry measurement.
